# Empowering limited-resource countries: collaborating with expert centers for diagnosis of primary ciliary dyskinesia

**DOI:** 10.3389/fmolb.2025.1547152

**Published:** 2025-03-20

**Authors:** Mine Yuksel Kalyoncu, Rim Hjeij, Muruvvet Yanaz, Aynur Gulieva, Merve Selcuk Balcı, Şeyda Karabulut, Neval Metin Cakar, Almala Pınar Ergenekon, Ela Erdem Eralp, Yasemin Gokdemir, Heymut Omran, Bülent Taner Karadag

**Affiliations:** ^1^ Department of Pediatric Pulmonology, Istanbul Kartal Dr.Lutfi Kirdar Education and Research Hospital, Istanbul, Türkiye; ^2^ Department of Pediatrics, University Hospital Münster, Münster, North Rhine-Westphalia, Germany; ^3^ Department of Pediatric Pulmonology, School of Medicine, Marmara University, Maltepe, Istanbul, Türkiye

**Keywords:** primary ciliary dyskinesia (PCD), international collaboration, low income countries, high speed video microscopy, rare disease, diagnostic challenges

## Abstract

**Introduction:**

Primary ciliary dyskinesia (PCD) is an autosomal recessive rare disease caused by alterations in ciliary structure and function. Without a unique gold standard diagnostic test, the European Respiratory Society and the American Thoracic Society recommend using various diagnostic techniques to improve accuracy. This study aimed to demonstrate the effectiveness of immunofluorescence (IF) analysis in the diagnosis of PCD cases with uncertain genetic results and to demonstrate the importance of international collaboration in the diagnosis of PCD.

**Methods:**

In collaboration with IF specialists at the University of Münster, individuals with inconclusive results in the Marmara University PCD panel consisting of the 22 most common genes and clinically suggestive of PCD were included in the study. IF imaging determined the subcellular localization of DNAH5 and GAS8 in respiratory epithelial cells. Nasal nitric oxide measurements, high-speed video microscopy (HSVM) analysis, and genetic analyses were performed.

**Results:**

19 patients were evaluated. The median age (25–75p) was 15 years (10–20 years) with 12 (63.2%) males. Three cases (15.7%) showed an absence of DNAH5, and one (5.3%) had a proximal distribution of DNAH5 in the ciliary axoneme. One case (5.3%) had cells without cilia, indicating a possible ciliogenesis defect. All individuals with abnormal IF analysis had a PICADAR score of 6 or above, and their cilia were immotile in HSVM.

**Discussion:**

Consistent with the IF finding suggesting a ciliogenesis defect, further genetic analysis revealed biallelic pathogenic variants in CCNO in the affected individual. The absence of DNAH5 in the respiratory epithelial cells of an individual carrying heterozygous pathogenic splice variants in DNAH5 suggests the need for further genetic analysis. This study underscores the importance of international collaboration in diagnosing rare diseases like PCD.

## 1 Introduction

Primary ciliary dyskinesia (PCD) (ORPHA:244) is a rare autosomal recessive genetic disorder characterized by abnormal movement of motile cilia (Frommer et al., 2015). It is a complex, heterogeneous, and multisystemic disease presenting with a range of clinical symptoms, including rhinosinusitis, middle ear disease, bronchiectasis (BE), and subfertility. Approximately half of the PCD-affected individuals exhibit situs abnormalities, and a subset of these individuals may also have associated congenital heart diseases or other organ abnormalities, particularly those with situs ambiguous or heterotaxy (Kennedy and Plant, 2014).

Diagnosing PCD can be challenging because there is no single gold standard test or method (O'Connor et al., 2021). The American Thoracic Society (ATS) and European Respiratory Society (ERS) recommend using a combination of diagnostic techniques to increase sensitivity and specificity (Shapiro et al., 2018; Lucas et al., 2017). Another barrier to accurate diagnosis is the complexity of the diagnostic methods, which require specialized equipment and trained staff (Leigh et al., 2011). Additionally, the lack of diagnostic devices in low-income countries further complicates the diagnosis process (Surdut et al., 2023).

In recent years, immunofluorescence (IF) staining has gained popularity as a diagnostic tool for PCD (Omran and Loges, 2009). This technique involves detecting ciliary proteins in respiratory cells using fluorescence or confocal microscopy and labeling them with antibodies targeting proteins such as outer (ODA) and inner (IDA) dynein arms, radial spokes (RS), dynein regulatory complex proteins (N-DRC), and central pair (CP) (Shapiro et al., 2018; Lucas et al., 2017). IF staining helps in recognizing the structure of the ciliary axoneme and has proven effective in confirming specific variants as pathogenic by identifying the location of protein compounds within the ciliary axoneme (Bhatt and Hogg, 2020). IF analysis is cheaper, faster, and more convenient than other ultrastructural analysis methods like transmission electron microscopy (TEM) (Shoemark et al., 2017). ERS Task Force experts agree that IF can be helpful in clinical settings (Lucas et al., 2017).

Our diagnostic approach for PCD primarily includes genetic analysis and nasal nitric oxide (NO) measurements. While nasal NO measurement is a valuable screening tool, its diagnostic utility is limited (Knowles et al., 2014; Raidt et al., 2022). Although the majority of PCD cases present with low nasal NO levels, certain genetic variants have been reported to exhibit normal or even elevated values (Knowles et al., 2014; Yiallouros et al., 2019; Collins et al., 2014). Consequently, this test cannot be relied upon as a conclusive diagnostic tool for PCD as it may not accurately identify all cases. To address this limitation, we introduced high-speed video microscopy (HSVM) in January 2023. A potential drawback of our diagnostic approach is that our PCD genetic panel comprises only 22 PCD-associated genes, making it challenging to diagnose patients with rare or novel variants. Consequently, we often require panels with more comprehensive genes or ultrastructural tests such as TEM and IF. Some patients exhibit highly suspicious clinical findings, but their genetic results show heterogeneous variations in PCD genes, necessitating ultrastructural tests like IF to confirm the presence of disease variants.

This study aims to diagnose individuals with highly suspicious clinical findings and unclear genetic results, emphasize the significance of IF analysis in diagnosing PCD, and highlight the benefits of collaborating with specialized centers in the diagnostic process.

## 2 Materials and methods

### 2.1 Subjects

Individuals with clinical symptoms and medical history suggestive of PCD and no pathological variants detected in PCD genetics performed using a targeted genetic panel, including the 22 most frequently identified PCD-associated genes (Table 1), were included in the study. Written informed consent was obtained from patients aged over 18 years and from the parents of patients under 18 years. Clinical and demographic data were collected from medical records. This study was approved by the Clinical Research Ethics Committee of Marmara University (Protocol No: 09.2023.111). Nasal NO measurements were conducted according to ERS recommendations using a CLD 88sp NO analyzer (ECO MEDICS, AG, Duerten, Switzerland) (Omran and Loges, 2009).

**TABLE 1:** Targeted genetic panel used for PCD in Marmara University.

Normal ultractructure	Outer dynein arm	Inner dynein arm and axonemal organization	Outer and inner dynein arm	Central apparatus and radial spoke	Absent or reduced cilia
*DNAH11*	*DNAH5*	*CCDC39*	*DNAAF1*	*RSPH9*	*FOXJ1*
*GAS2L2*	*DNAI1*	*CCDC40*	*DNAAF2*	*RSPH4A*	
	*DNAI2*		*DNAAF3*	*HYDIN*	
	*DNAL1*		*DNAAF4*	*SPEF2*	
	*NME8*		*DNAAF5*		
	*DNAH9*		*CCDC103*		
			*LRRC6*		

### 2.2 Immunofluorescence analysis

To obtain respiratory epithelial cells, a transnasal brush biopsy (Cytobrush Plus; Medscand Medical, Malmö, Sweden) was performed (Omran and Loges, 2009). The cells were suspended in cell culture medium (RPMI), spread onto glass slides, and air-dried at the outpatient clinics of Marmara University. These samples were then sent to the IF laboratories at the University of Münster between November and December 2022. IF analysis was performed in a cohort of 19 PCD-suspected individuals and five healthy controls at the University of Münster. The cells were treated with 4% paraformaldehyde, 0.2% Triton X-100, and 1% skim milk before incubation with primary antibodies (3–4 h) and secondary antibodies (30 min) at room temperature (Omran and Loges, 2009). Individuals were analyzed using antibodies against component proteins for the ODAs (*DNAH5*) and the nexin-dynein regulatory complex (*GAS8*) of the ciliary axoneme (Omran and Loges, 2009). Monoclonal Mouse anti-*DNAH5* and polyclonal rabbit anti-*GAS8* (HPA041311) primary antibodies were used for double labeling at a 1:500 dilution (Omran and Loges, 2009). Mouse monoclonal anti-DNAH5 antibody was generated as previously described (Omran et al., 2008). Goat Anti-mouse Alexa Fluor 488 and anti-rabbit Alexa Fluor 546 secondary antibodies were used as 1:1000 dilution. To visualize cell nuclei, DNA was stained with Hoechst 33342 (Sigma). High-resolution fluorescence images were were taken using a Zeiss Axiovert 200 equiped with ApoTome.2 using a PlanApo 63X/1.4NA oil objective. Images were taken using a AxionCam712 mono and processed with ZEN2 Blue software. Figures were prepared with Adobe Creative Suite 4 (Omran and Loges, 2009; Dougherty et al., 2016).

### 2.3 High-speed video microscopy analysis

After IF staining was completed in Münster, HSVM analyses were performed as an additional diagnostic tool at Marmara University between January 2023 and March 2023. Nasal epithelial cells were obtained by using the nasal brushing technique from patients (Dougherty et al., 2016). Participants were recruited if they had not taken nasal steroids or decongestants for at least 4 weeks and had not developed symptoms of acute respiratory tract infection for at least 4 weeks. Ciliated cells placed in a pre-warmed culture medium (RPMI 1640-Medium) at a temperature of 37°C^17^. The cells were subsequently equilibrated to the optimal temperature of 37°C on a heater plate (Tpi-TSX, Tokai, Japan). The frequency of ciliary movement was measured using HSVM with the Sisson-Ammons Video Analysis software (SAVA, MI, United States). The measurements were taken with an inverted phase-contrast Nikon Eclipse TS100 microscope (Nikon, Japan) equipped with a ×40 objective and linked to a digital high-speed video camera (Basler acA1300-200um, Germany). The digital image sampling was set at 640 × 480 pixels and a frame rate of 120–150 frames per second (fps) for a duration of one minute, with intervals of 15 s. The ciliary beat was analyzed from both top and side views using real-time and slow-motion replay (Raidt et al., 2014). Ciliary beat patterns (CBP) were defined as “normal, virtually immotile, stiff beating with a reduced amplitude, circular gyrating motion” and ciliary beat frequency were measured (Raidt et al., 2014). The results obtained in HSVM could not be confirmed through air–liquid interface (ALI) culture as it is not available at our center.

### 2.4 Statistical analysis

Statistical analysis was conducted using IBM^®^ SPSS^®^ Statistics Version 20 software. For descriptive analysis, normally distributed data were expressed as mean ± SD, while non-normally distributed data were expressed as median [interquartile range (IQR)]. Statistical significance was set at p < 0.05.

## 3 Results

Nineteen PCD-affected individuals were included in this study. The median age (25th-75th percentile) was 15 years (10–20 years) with 12 (63.2%) individuals being male (Table 2).

**TABLE 2:** Characteristics of PCD-suspected individuals (n:19).

ID	Gender/Age (year)	Cons	Nasal NO (nL/dk)	Situs inversus totalis	BE	nRDS	Chronic rhinitis	Recurrent otitis and sinusitis	PICADAR	Genetic variant	Variant analyses	IF staining for DNAH5 and GAS8	HSVM findings (37°)
AG	M/15	+	15	−	+	+	+	−	7	*RSPH4A hom*. *c.1105G>C (p.Ala369Pro)*	Pathogenic moderate in Varsome VUS in Clinvar and Franklin ACMG classification	Normal	Abnormal beat pattern
TS	F/17	+	0	−	+	−	+	+	6	*HYDIN hom. exon 5–16 deletion* *CCDC40 het. c.2893 G>A (p.Ala965Thr)* *DNAAF4 het. c.408 T>G (p.lle136Met)*	- Benign in Varsome, Clinvar and Franklin ACMG classification Benign moderate in Varsome Not available on Clinvar VUS in Franklin ACMG classification	Normal	Abnormal beat pattern
HAA	M/11	+	90	−	−	+	+	−	7	*CCNO hom*. c.248_252dup p(Gly85Cysfs*11)	Pathogenic on Franklin ACMG classification and Clinvar	No ciliated cells	Immotile
HNA	F/13	−	449	−	−	−	+	−	3	*DNAH11 het c.12632C>T (p.Pro4211Leu)* *DNAH11 het. c.13399G>A (p.Val4467Met)*	Likely benign in Varsome VUS in Franklin ACMG classification. VUS on Clinvar Strong benign in Varsome Likely benign in Franklin ACMG classification Conflicting classifications of pathogenicity Uncertain significance (2); Likely benign (2) on Clinvar	Normal	Normal beat pattern
Kİ	M/16	+	414	−	+	−	+	−	3	DNAH11 het? *c.9935 A>T (p.Asp3312Val)* *DNAH11 het c.9435 G>A (p.Thr3145Thr)* *CCDC39 het. c.808 G>A (p.Glu270Lys)* *CCDC40 het. c.1607 G>A (p.Arg536His)*	Likely benign in Franklin ACMG classification and Clinvar Benign in Franklin ACMG classification. Conflicting classifications of pathogenicity: Uncertain significance (1); Benign (3); Likely benign (2) on Clinvar Likely benign in Varsome VUS in Franklin ACMG classification Likely benign in Varsome VUS in Franklin ACMG classification. Not available on Clinvar	Normal	Normal beat pattern
OS	M/22	−	595	−	+	−	−	−	2	*DNAH5 het. c.7748 G>T (p.Gly2583Val)* *DNAH5 het. c.1206 T>A (p.Asn402Lys)*	VUS in Varsome, Franklin ACMG classification and Clinvar Benign moderate in Varsome and Franklin ACMG classification Conflicting classifications of pathogenicity Uncertain significance (6); Likely benign (2) in Clinvar	Normal	Abnormal beat pattern
MA	F/14	+	387	+	−	−	−	−	6	*NME8 het. c.82 G>T (p.Gly28Cys)* *FOXJ1 het. c.165 G>C (p.Gly55Gly)*	Likely pathogenic in Varsome VUS in ClinVar and Franklin ACMG classification VUS in Franklin ACMG classification Not available on Clinvar	Normal	Abnormal beat pattern
ÖG	M/20	+	108	+	+	+	+	+	11	*CCDC40 het. c.1564 G>A (p.Val522Met)* *DNAAF4 het. c.1070 T>C (p.Ile357Thr)*	Strong benign in Varsome and Franklin ACMG classification VUS on Clinvar Strong benign in Varsome VUS in Franklin ACMG classification Not available on Clinvar	DNAH5 absent GAS8 normal	Immotile
ÖB	M/20	+	11	−	+	−	−	+	3	GAS2L2; het. c.2597 G>A (p.Trp866*)	Strong benign in Varsome VUS in Franklin ACMG classification. Not available on Clinvar	Normal	Abnormal beat pattern
İT	M/7	+	609	−	−	−	−	−	2	*DNAAF3; het. c.521T>C (p.Leu174Pro)*	Moderate benign in Varsome VUS in Franklin ACMG classification. Not available on Clinvar	Normal	Abnormal beat pattern
EEE	M/10	−	169	−	−	−	+	−	3	*HYDIN; het. c.4193A>G (p.Gln1398Arg)* *DNAH9; het. c.4902C>T (p.Asp1634Asp)*	Likely benign in Varsome VUS in Franklin ACMG classification. Not available on Clinvar VUS in Franklin ACMG classification. Not available on Clinvar	Normal	NA
ÖY	M/11	+	NA	−	−	+	+	−	7	*DNAH5 het. c. 9106-1G>A (p.Gln512Gln)* *HYDIN het c.4021 C>G(p.Pro1341Ala)* *DNAI2 het c.1538 C>T (p.Ala513Val)*	Pathogenic in Varsome, Franklin ACMG classification and Clinvar VUS in Varsome and Franklin ACMG classification Not available on Clinvar Benign strong in Varsome and Franklin ACMG classification Conflicting classifications of pathogenicity Uncertain significance (2); Likely benign (4) on Clinvar	DNAH5 absent GAS8 normal	NA
YAA	M/8	−	731	−	−	+	+	+	6	*DNAH11 het c.10768C>G (p.Pro3590Ala)* *RSPH4A het c.1377 G>T (p.Lys459Asn)*	Strong benign in Varsome and Clinvar VUS in Franklin ACMG classification Benign moderate in Varsome VUS in Franklin ACMG classification	Normal	Immotile
BMT	F/7	−	NA	+	−	+	−	−	10	*No known mutation*	-	Normal	Abnormal beat pattern
CB	F/17	+	38	+	+	+	+	+	11	*No known mutation*	-	DNAH5 absent GAS8 normal	Immotile
İİ	F/22	+	168	−	+	−	−	−	3	*No known mutation*	-	Normal	Abnormal beat pattern
İK	F/21	+	30	+	+	+	−	−	10	*No known mutation*	-	DNAH5 proximal GAS8 normal	Immotile
EAT	M/9	−	668	+	-	+	−	−	10	*No known mutation*	-	Normal	Abnormal beat pattern
SK	M/22	-	543	−	+	*-*	+	−	3	*No known mutation*	-	Normal	Abnormal beat pattern

NA, not available; Cons, consanguinity; nRDS, neonatal respiratory distress syndrome; BE, bronchiectasis.

We performed IF analysis in controls and PCD-affected individuals targeting DNAH5 (structural component of ODAs) and GAS8 (structural component of N-DRCs). In unaffected controls, DNAH5 and GAS8 localize to the axonemal length. However, we detected an abnormal localization of DNAH5 in five cases (Table 2). DNAH5 was completely absent in three cases (15.7%) (CB, ÖG, and ÖY) who shared clinical characteristics: neonatal respiratory distress, chronic rhinitis, recurrent otitis and sinusitis. All had immotile cilia on HSVM. CB and ÖG presented with situs inversus totalis and BE, while ÖY lacked these. Genetic analysis revealed no pathogenic variants in CB and ÖG, whereas ÖY carried a heterozygous *DNAH5* splice variant (c.9106-1G>A). Nasal NO levels varied, with CB showing lower values (38.94) compared to ÖG (108.23 nL/min).

In only one case (IK) (5.3%), IF staining showed proximal distribution of DNAH5 in the ciliary axoneme. This patient, in contrast to numerous PCD cases, did not exhibit chronic rhinitis, recurrent otitis, sinusitis, or situs inversus totalis; however, the patient presented with a history of neonatal respiratory distress and BE. Nasal NO levels were low (30.68 nL/min), genetic analysis did not identify a pathogenic variant, and HSVM findings demonstrated immotile cilia.

**FIGURE 1:**
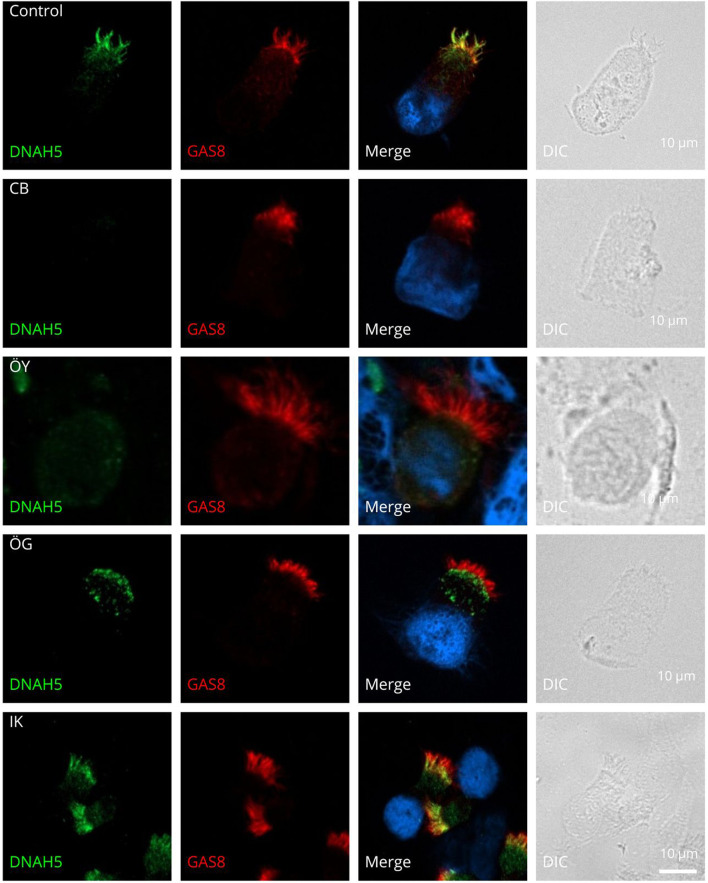
Outer dynein arm defects by IF. Respiratory cilia double-labeled with antibodies directed against DNAH5 (green) and GAS8 (red) show colocalization of DNAH5 with GAS8 along the cilia from healthy controls. In cells of three PCD-affected individuals CB, OY and OG, DNAH5 is absent from the ciliary axonemes and is proximal in one individual IK. Nuclei were stained with Hoechst33342 (blue). Scale bars represent 10 μm.

One individual (HAA) (5.3%) showed cells without cilia in IF staining. This patient presented with a history of neonatal respiratory distress, chronic rhinitis, and recurrent otitis and sinusitis. In contrast to typical PCD cases, the patient did not exhibit situs inversus totalis or BE, and nasal NO levels were relatively elevated (90.27 nL/min). Initial genetic analysis did not detect any pathogenic variants. Subsequently, whole exome sequencing (WES) was performed. However, after the patient submitted a sample for IF staining, WES analysis was completed, revealing a biallelic pathogenic *CCNO* variant (c.248_252dup p (Gly85Cysfs*11). Figure 1 illustrates examples of the absence or abnormal localization of DNAH5 in four individuals with confirmed PCD.

In two individuals (AG and TS), IF results were normal despite low nasal NO and an abnormal beat pattern observed through HSVM (Table 2). One individual with situs inversus (BMT), a PICADAR score of 10, and an abnormal beat pattern detected by HSVM also had normal IF results (Table 2). WES analysis was performed at the time of IF sampling for AG; however, the results were not yet available. Following IF analysis, a homozygous RSPH4A c.1105G>C (p.Ala369Pro) pathogenic variant was identified.

After the implementation of HSVM in our center in collaboration with Münster, the remaining patients were recalled for HSVM assessment. WES analysis were planned for cases with abnormal HSVM results, low nasal NO measurements, and a PICADAR score above 5.

All individuals with abnormal IF analysis had a PICADAR score greater than 7. The cilia of all individuals with abnormal IF were immotile according to HSVM.

Parental consanguinity was observed in 100% of the individuals, and all had neonatal respiratory distress. Table 2 summarizes the clinical and demographic features, along with HSVM findings, of the patients with abnormal IF results.

## 4 Discussion

In this study, we successfully diagnosed PCD using IF analysis in individuals with inconclusive results from multiple diagnostic tools, including genetic testing, HSVM, and nasal NO measurement. The addition of IF analysis significantly enhanced our diagnostic capabilities. Furthermore, through international collaboration, HSVM has been introduced at our institution, resulting in notable advancements in the diagnosis of PCD.

At our center, current practice involves using nasal NO measurements and genetic testing to evaluate suspected PCD cases. Genetic panels now offer broader gene coverage and are more cost-effective than WES (Platt et al., 2021). Our panel includes the 22 most frequently observed PCD-related genes; however, limitations persist, such as incomplete genetic diagnoses due to unknown genes, variants of uncertain significance, or single heterozygous variants not included in the panel. As a result, many individuals with highly suggestive clinical features remain without genetic confirmation of PCD.

Diagnosing PCD remains complex and challenging due to the absence of disease-specific symptoms, screening tests, and a single gold-standard diagnostic method. Additionally, PCD centers are rare, and the lack of a standardized diagnostic algorithm further complicates diagnosis and awareness efforts (Behan et al., 2016a; Rubbo and Lucas, 2017). Current guidelines recommend a combination of diagnostic tools, but algorithms vary significantly between regions (Shapiro et al., 2018; Lucas et al., 2017). For instance, the ERS recommends HSVM as the first diagnostic test, while the ATS suggests extended genetic panel testing (Shapiro et al., 2018; Lucas et al., 2017). In our country, limited diagnostic resources have resulted in an approach similar to the ATS algorithm, where genetic testing follows nasal NO screening. However, genetic analysis is often time-consuming and limited in scope. To address these limitations, we introduced HSVM into our diagnostic repertoire through international collaboration with the University of Münster.

International collaborations have demonstrated effectiveness in diagnosing and managing rare or complex conditions in resource-limited settings. Such partnerships have facilitated the diagnosis of inherited thrombocytopenias in Argentina and improved the understanding of barriers to pediatric cancer diagnosis in Western Kenya (Glembotsky et al., 2012; Severance et al., 2022). However, examples of collaboration for PCD diagnosis remain limited (Rumman et al., 2023). Our study aimed to address diagnostic uncertainties for individuals with inconclusive results through IF analysis, enabling identification of underlying causes in a subset of cases. Moreover, our institution has successfully integrated HSVM into its diagnostic protocol. Similar to our findings, Rumman et al. reported that 68 of 464 clinically suspected PCD patients were diagnosed through international collaboration (Rumman et al., 2023).

Previous studies have reported cases of PCD where patients exhibited normal IF results despite a definitive diagnosis (Biebach et al., 2022; Shapiro and Leigh, 2017). Additionally, IF analysis may be insufficient for diagnosing PCD individuals with central pair (CP) defects, as the absence of the CP-associated *SPEF2* protein has been noted in *HYDIN* mutant cells (Cindric et al., 2020). In our study, three individuals with high clinical suspicion of PCD had abnormal nasal NO levels and HSVM findings but normal IF results. This discrepancy may arise from genes associated with normal ultrastructure or the need for additional staining with specific IF antibodies. Although IF analysis is widely used to confirm the absence of proteins caused by genetic mutations, its diagnostic efficacy is constrained by the availability of validated antibodies and its reported low sensitivity in PCD diagnosis (Shoemark et al., 2017; Baz-Redon et al., 2020). Furthermore, as novel genes and proteins associated with PCD continue to be identified, the IF antibody panel will require expansion (Knowles and Leigh, 2017).

To determine the likelihood of PCD, researchers have proposed various predictive tools (Martinu et al., 2021). Among these, the PICADAR score has emerged as a widely utilized tool, particularly in low-resource settings (Behan et al., 2016b; Rubin, 1988). A PICADAR score of 10 corresponds to a probability of 92.6% for PCD, while a score >5 demonstrates good sensitivity and specificity for clinically suspected cases (Behan et al., 2016b). However, the optimal cut-off may vary; one study suggested a score of 2 as the best discriminative value (Rademacher et al., 2017). In a Japanese cohort, most patients had PICADAR scores ≤5, indicating that individuals with chronic wet cough, even with low scores, should still be evaluated for PCD (Chiyonobu et al., 2022). In our cohort, all five individuals had PICADAR scores ≥7. Notably, all three patients with normal IF results had PICADAR scores of ≥6 and low nasal NO levels. Further studies involving larger populations are needed to better assess the sensitivity and specificity of the PICADAR score.

The primary limitation of this study is its small sample size. Additionally, the absence of a reliable reference standard for assessing IF accuracy presents a significant challenge. For benchmarking purposes, we utilized the ERS Task Force criteria to classify confirmed and highly probable PCD cases (Lucas et al., 2017).

In conclusion, this study underscores the critical role of international collaboration in diagnosing rare diseases like PCD, which often require expertise available only in specialized centers. IF analysis is not routinely available at our institution; however, collaboration with the University of Münster enabled us to diagnose individuals in whom conventional methods were inconclusive. Such partnerships between resource-limited settings and specialized centers can significantly enhance diagnostic capabilities, particularly when disease prevalence is lower than anticipated. Furthermore, establishing international registry systems that include data from developing countries will contribute to improved diagnosis and management of PCD. Collaboration and shared expertise are key to addressing the diagnostic challenges associated with rare diseases.

## Data Availability

The original contributions presented in the study are included in the article/Supplementary Material, further inquiries can be directed to the corresponding authors.

## References

[B1] Baz-Redon N. Rovira-Amigo S. Fernandez-Cancio M. Castillo-Corullon S. Cols M. Caballero-Rabasco M. A. (2020). Immunofluorescence analysis as a diagnostic tool in a Spanish cohort of patients with suspected primary ciliary dyskinesia. J. Clin. Med. 9 (11), 3603. 10.3390/jcm9113603 33182294 PMC7695268

[B2] Behan L. Dimitrov B. D. Kuehni C. E. Hogg C. Carroll M. Evans H. J. (2016b). Picadar: a diagnostic predictive tool for primary ciliary dyskinesia. Eur. Respir. J. 47 (4), 1103–1112. 10.1183/13993003.01551-2015 26917608 PMC4819882

[B3] Behan L. Dunn Galvin A. Rubbo B. Masefield S. Copeland F. Manion M. (2016a). Diagnosing primary ciliary dyskinesia: an international patient perspective. Eur. Respir. J. 48 (4), 1096–1107. 10.1183/13993003.02018-2015 27492837 PMC5045441

[B4] Bhatt R. Hogg C. (2020). Primary ciliary dyskinesia: a major player in a bigger game. Breathe (Sheff). 16 (2), 200047. 10.1183/20734735.0047-2020 33304404 PMC7714554

[B5] Biebach L. Cindric S. Koenig J. Aprea I. Dougherty G. W. Raidt J. (2022). Recessive mutations in cfap74 cause primary ciliary dyskinesia with normal ciliary ultrastructure. Am. J. Respir. Cell Mol. Biol. 67 (3), 409–413. 10.1165/rcmb.2022-0032LE 36047773 PMC9447143

[B6] Chiyonobu K. Xu Y. Feng G. Saso S. Ogawa S. Ikejiri M. (2022). Analysis of the clinical features of Japanese patients with primary ciliary dyskinesia. Auris Nasus Larynx 49 (2), 248–257. 10.1016/j.anl.2021.08.003 34454779

[B7] Cindric S. Dougherty G. W. Olbrich H. Hjeij R. Loges N. T. Amirav I. (2020). Spef2-and hydin-mutant cilia lack the central pair-associated protein spef2, aiding primary ciliary dyskinesia diagnostics. Am. J. Respir. Cell Mol. Biol. 62 (3), 382–396. 10.1165/rcmb.2019-0086OC 31545650

[B8] Collins S. A. Gove K. Walker W. Lucas J. S. (2014). Nasal nitric oxide screening for primary ciliary dyskinesia: systematic review and meta-analysis. Eur. Respir. J. 44 (6), 1589–1599. 10.1183/09031936.00088614 25323224

[B9] Dougherty G. W. Loges N. T. Klinkenbusch J. A. Olbrich H. Pennekamp P. Menchen T. (2016). Dnah11 localization in the proximal region of respiratory cilia defines distinct outer dynein arm complexes. Am. J. Respir. Cell Mol. Biol. 55 (2), 213–224. 10.1165/rcmb.2015-0353OC 26909801 PMC4979367

[B10] Frommer A. Hjeij R. Loges N. T. Edelbusch C. Jahnke C. Raidt J. (2015). Immunofluorescence analysis and diagnosis of primary ciliary dyskinesia with radial spoke defects. Am. J. Respir. Cell Mol. Biol. 53 (4), 563–573. 10.1165/rcmb.2014-0483OC 25789548 PMC5306451

[B11] Glembotsky A. C. Marta R. F. Pecci A. De Rocco D. Gnan C. Espasandin Y. R. (2012). International collaboration as a tool for diagnosis of patients with inherited thrombocytopenia in the setting of a developing country. J. Thromb. Haemost. 10 (8), 1653–1661. 10.1111/j.1538-7836.2012.04805.x 22672365

[B12] Kennedy M. P. Plant B. J. (2014). Primary ciliary dyskinesia and the heart: cilia breaking symmetry. Chest 146 (5), 1136–1138. 10.1378/chest.14-0722 25367461

[B13] Knowles M. R. Leigh M. W. (2017). Primary ciliary dyskinesia diagnosis. Is color better than black and white? Am. J. Respir. Crit. Care Med. 196 (1), 9–10. 10.1164/rccm.201702-0426ED 28665204

[B14] Knowles M. R. Ostrowski L. E. Leigh M. W. Sears P. R. Davis S. D. Wolf W. E. (2014). Mutations in rsph1 cause primary ciliary dyskinesia with a unique clinical and ciliary phenotype. Am. J. Respir. Crit. Care Med. 189 (6), 707–717. 10.1164/rccm.201311-2047OC 24568568 PMC3983840

[B15] Leigh M. W. O'Callaghan C. Knowles M. R. (2011). The challenges of diagnosing primary ciliary dyskinesia. Proc. Am. Thorac. Soc. 8 (5), 434–437. 10.1513/pats.201103-028SD 21926395 PMC3209576

[B16] Lucas J. S. Barbato A. Collins S. A. Goutaki M. Behan L. Caudri D. (2017). European respiratory society guidelines for the diagnosis of primary ciliary dyskinesia. Eur. Respir. J. 49 (1), 1601090. 10.1183/13993003.01090-2016 27836958 PMC6054534

[B17] Martinu V. Borek-Dohalska L. Varenyiova Z. Uhlik J. Capek V. Pohunek P. (2021). Evaluation of a clinical index as a predictive tool for primary ciliary dyskinesia. Diagn. (Basel) 11 (6), 1088. 10.3390/diagnostics11061088 PMC8232329 34198708

[B18] O'Connor M. G. Horani A. Shapiro A. J. (2021). Progress in diagnosing primary ciliary dyskinesia: the north american perspective. Diagn. (Basel) 11 (7), 1278. 10.3390/diagnostics11071278 PMC8304305 34359360

[B19] Omran H. Kobayashi D. Olbrich H. Tsukahara T. Loges N. T. Hagiwara H. (2008). Ktu/pf13 is required for cytoplasmic pre-assembly of axonemal dyneins. Nature 456 (7222), 611–616. 10.1038/nature07471 19052621 PMC3279746

[B20] Omran H. Loges N. T. (2009). Immunofluorescence staining of ciliated respiratory epithelial cells. Methods Cell Biol. 91, 123–133. 10.1016/S0091-679X(08)91007-4 20409784

[B21] Platt C. D. Zaman F. Bainter W. Stafstrom K. Almutairi A. Reigle M. (2021). Efficacy and economics of targeted panel versus whole-exome sequencing in 878 patients with suspected primary immunodeficiency. J. Allergy Clin. Immunol. 147 (2), 723–726. 10.1016/j.jaci.2020.08.022 32888943 PMC7870529

[B22] Rademacher J. Buck A. Schwerk N. Price M. Fuge J. Welte T. (2017). Nasal nitric oxide measurement and a modified picadar score for the screening of primary ciliary dyskinesia in adults with bronchiectasis. Pneumologie 71 (8), 543–548. 10.1055/s-0043-111909 28783864

[B23] Raidt J. Krenz H. Tebbe J. Grosse-Onnebrink J. Olbrich H. Loges N. T. (2022). Limitations of nasal nitric oxide measurement for diagnosis of primary ciliary dyskinesia with normal ultrastructure. Ann. Am. Thorac. Soc. 19 (8), 1275–1284. 10.1513/AnnalsATS.202106-728OC 35202559

[B24] Raidt J. Wallmeier J. Hjeij R. Onnebrink J. G. Pennekamp P. Loges N. T. (2014). Ciliary beat pattern and frequency in genetic variants of primary ciliary dyskinesia. Eur. Respir. J. 44 (6), 1579–1588. 10.1183/09031936.00052014 25186273

[B25] Rubbo B. Lucas J. S. (2017). Clinical care for primary ciliary dyskinesia: current challenges and future directions. Eur. Respir. Rev. 26 (145), 170023. 10.1183/16000617.0023-2017 28877972 PMC9489029

[B26] Rubin B. K. (1988). Immotile cilia syndrome (primary ciliary dyskinesia) and inflammatory lung disease. Clin. Chest Med. 9 (4), 657–668. 10.1016/s0272-5231(21)00590-6 3069296

[B27] Rumman N. Fassad M. R. Driessens C. Goggin P. Abdelrahman N. Adwan A. (2023). The palestinian primary ciliary dyskinesia population: first results of the diagnostic and genetic spectrum. ERJ Open Res. 9 (2), 00714. 10.1183/23120541.00714-2022 37077557 PMC10107064

[B28] Severance T. S. Njuguna F. Olbara G. Kugo M. Langat S. Mostert S. (2022). An evaluation of the disparities affecting the underdiagnosis of pediatric cancer in western Kenya. Pediatr. Blood Cancer 69 (10), e29768. 10.1002/pbc.29768 35593641

[B29] Shapiro A. J. Davis S. D. Polineni D. Manion M. Rosenfeld M. Dell S. D. (2018). Diagnosis of primary ciliary dyskinesia. An official american thoracic society clinical practice guideline. Am. J. Respir. Crit. Care Med. 197 (12), e24–e39. 10.1164/rccm.201805-0819ST 29905515 PMC6006411

[B30] Shapiro A. J. Leigh M. W. (2017). Value of transmission electron microscopy for primary ciliary dyskinesia diagnosis in the era of molecular medicine: genetic defects with normal and non-diagnostic ciliary ultrastructure. Ultrastruct. Pathol. 41 (6), 373–385. 10.1080/01913123.2017.1362088 28915070 PMC6047068

[B31] Shoemark A. Frost E. Dixon M. Ollosson S. Kilpin K. Patel M. (2017). Accuracy of immunofluorescence in the diagnosis of primary ciliary dyskinesia. Am. J. Respir. Crit. Care Med. 196 (1), 94–101. 10.1164/rccm.201607-1351OC 28199173 PMC5519960

[B32] Surdut S. P. van der Merwe E. Goussard P. Urban M. F. (2023). Which side are they on? Diagnosing primary ciliary dyskinesias in low- or middle-income countries: a review and case series. Afr. J. Thorac. Crit. Care Med. 29 (3), 131–138. 10.7196/AJTCCM.2023.v29i3.425 PMC10646753 38028243

[B33] Yiallouros P. K. Kouis P. Pirpa P. Michailidou K. Loizidou M. A. Potamiti L. (2019). Wide phenotypic variability in rsph9-associated primary ciliary dyskinesia: review of a case-series from Cyprus. J. Thorac. Dis. 11 (5), 2067–2075. 10.21037/jtd.2019.04.71 31285900 PMC6588774

